# Effect of geometric variations on tibiofemoral surface and post-cam design of normal knee kinematics restoration

**DOI:** 10.1186/s40634-018-0167-z

**Published:** 2018-12-21

**Authors:** Yong-Gon Koh, Ji-Hoon Nam, Kyoung-Tak Kang

**Affiliations:** 1 Joint Reconstruction Center, Department of Orthopaedic Surgery, Yonsei Sarang Hospital, 10 Hyoryeong-ro, Seocho-gu, Seoul, 06698 Republic of Korea; 2 Department of Mechanical Engineering, Yonsei University, 50 Yonsei-ro, Seodaemun-gu, Seoul, 03722 Republic of Korea

**Keywords:** Patient-specific implant, Post-cam design, Conformity, Total knee arthroplasty, Finite element analysis

## Abstract

**Background:**

Restoration of natural knee kinematics for a designed mechanism in knee implants is required to achieve full knee function in total knee arthroplasty (TKA). In different posterior-stabilized TKAs, there are wide variations in tibiofemoral surfaces and post-cam design. However, it is not known whether these design variations preserve natural knee kinematics. The purpose of this study was to determine the most appropriate tibiofemoral surface and post-cam designs to restore natural knee kinematics of the TKA.

**Methods:**

A subject-specific finite element knee modal was used to evaluate tibiofemoral surface and post-cam design. Three different posts in convex, straight, and concave geometries were considered with a fixed circular cam design in this study. In addition, this post-cam design was applied to three different surface conformities for conforming, medial pivot, and subject anatomy mimetic tibiofemoral surfaces. We evaluated the femoral rollback, internal-external rotation, and quadriceps muscle force under a deep-knee-bend condition.

**Results:**

The three different tibiofemoral conformities showed that the convex post provided the most natural-knee-like femoral rollback. This was also observed in internal rotation. In surface conformity, subject anatomy mimetic tibiofemoral surfaces showed the most natural -knee-like kinematics and quadriceps force.

**Conclusions:**

This study confirmed that convex post design and subject anatomy mimetic tibiofemoral surfaces provided the most natural-knee-like kinematics. This study suggested that post-cam design and tibiofemoral surface conformity should be considered in conventional and customized TKA.

## Background

In the 1970s, posterior-stabilized (PS) total knee arthroplasty (TKA) designs were introduced as an alternative to existing cruciate-retaining (CR) designs (Insall et al., 1983). The tibial post and femoral cam mechanism take a role of the posterior cruciate ligament providing femoral roll-back with knee flexion and preventing posterior tibial subluxation during knee flexion (Watanabe et al., 2017).

In biomechanical engineering point of view, the use of a post-cam mechanism is a straightforward choice. For all conceivable mechanisms, a post to the polyethylene (PE) insert and a cam to the femoral component is a relatively simple and functionally robust solution (Arnout et al., 2015). It was reported in in vivo and in vitro studies that the kinematic patterns of PS high-flex knee implants closely resemble the intact knee joint motion (Argenson et al., 2005; G. Li et al., 2004). The use of a post-cam mechanism in PS TKA is intended to facilitate this femoral rollback phenomenon. In addition, despite its importance, the post cam mechanism in some designs has caused several problems. Wear, deformity, and post breakage have been reported because of the high stress on the post (Kumar et al., 2015; Lachiewicz, 2011; Mauerhan, 2003). Although the PS with an intercondylar cam-post is treated as a generic style of TKA, there are many available designs for both articular surface conformity in the frontal and sagittal radii of the bearing surfaces and in the configuration with a post-cam design mechanism (Walker et al., 2014).

Because kinematic changes with respect to articular surface conformity and it could affect to engagement in post-cam mechanism (Koh et al., 2018). In recent years, natural kinematic behavior in a TKA was attempted with the goal of improving functional performance. The Medial Pivot design from Wright Manufacturing Co. consisted of a tibial component with a more dished medial side and a shallow lateral side (Blaha, 2004). Another design used a dished medial tibial surface and a convex lateral surface having post-cam (Koh et al., 2018; Victor & Bellemans, 2006). In developing a new design, it is ideal to evaluate the kinematics throughout the design process before clinical application. The restoration of kinematics after TKA has been of great concern. Many studies have investigated the effects of TF articular surface conformity and post-cam design on kinematics to restore natural knee kinematics (Argenson et al., 2005; Lin et al., 2011; Pandit et al., 2005; Walker et al., 2009). However, most studies investigated TF articular surface conformity and post-cam design separately to restore natural knee kinematics (Argenson et al., 2005; Lin et al., 2011; Pandit et al., 2005; Walker et al., 2009). The two main approaches are computational modeling and experimental study (Fitzpatrick et al., 2012; Liu et al., 2012; Walker et al., 2015; Walker et al., 2014). Computational modeling has the advantage that many different activity scenarios can be simulated, and different design variations can be evaluated (Walker et al., 2015). Experimental study can include implantations into knee specimens and also provide direct observation of the mechanics, often providing insight into potential problems or design modifications (Walker et al., 2015). A benchmark against which to evaluate the replacement in many functional conditions is necessary. However, experimental studies using cadavers are usually performed on the elderly; thus, if forces are repeatedly applied under loads, loosening between the specimen and device and some attenuation of the tissue itself may occur (Kang et al., 2017b). A computational knee joint model eliminates some of the disadvantages of in vitro studies, such as limitations from specimens under quasistatic loading conditions (Kang et al., 2017b ). In addition, the advantage of computational simulations involving the use of a single subject is that the effects of the post-cam design and conformity for a TF articular surface within a subject are determined without the effects of variables such as weight, height, bony geometry, ligament properties, and component size (Kang, Koh, et al., 2018a). Computational models with validation can be considered an effective methodology in parametric analyses and population-based clinical studies (Kiapour et al., 2014). In addition, no studies have investigated natural knee kinematics with respect to different post-cam designs and articular surface conformities using computational simulation.

Therefore, the purpose of this study was to evaluate the magnitude of the kinematic differences between different PS designs, followed by a determination of the design characteristics that would more closely reproduce the kinematics of the normal knee using finite element (FE) model. Accordingly, we first applied three different posts in convex, straight, and concave geometries with a fixed circle cam in a post-cam design. Second, three different conformities including conforming design, medial pivot design, and anatomy mimetic tibiofemoral surface were applied to the three different post-cam designs. Finally, femoral rollback, internal external rotation, and quadriceps force were evaluated in nine different PS TKA designs under a deep-knee-bend condition. We hypothesized that circle cam and convex post in anatomy mimetic tibiofemroal surface shows natural knee like biomechanics.

## Methods

### Development of normal knee model

**Fig. 1:**
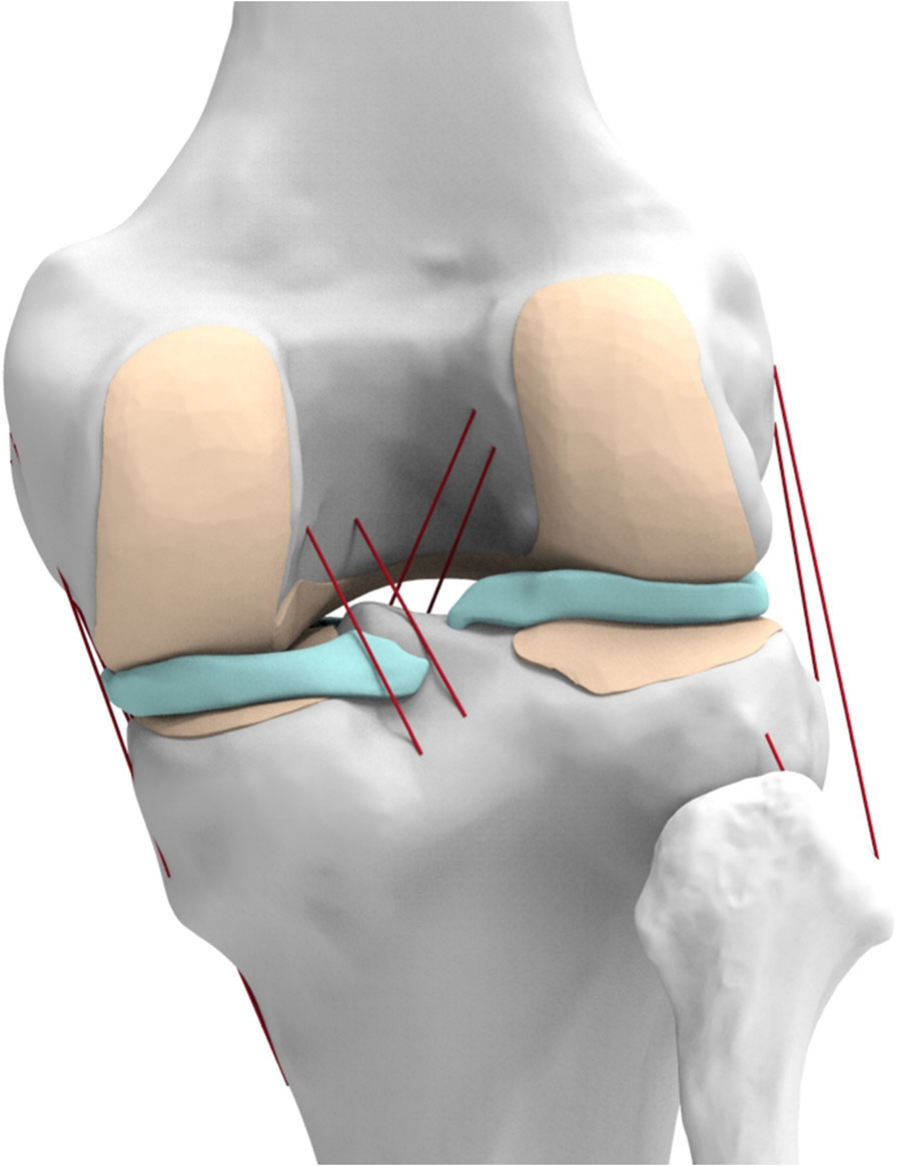
3D FE knee joint model developed from imaging data used in this study

A previously developed normal knee finite element (FE) model was used in this study (Kang et al., 2016a; Kang et al., 2017b; Kang et al., 2018b; Kim et al., 2015). An anatomically accurate FE model of the lower extremity was developed from the imaging data of a healthy, skeletally mature young male athlete without a history of knee injury (Kang et al., 2016a; Kang et al., 2017b; Kang et al., 2018b; Kim et al., 2015). The model includes bony structures of the lower extremity in addition to soft tissue details of the patellofemoral (PF) and tibiofemoral (TF) knee joints. The model includes major ligaments, trans-knee muscles, articular cartilage, and menisci (Fig. 1).

A 3D knee joint model was developed from computed tomography (CT) and magnetic resonance imaging (MRI) data followed by 3Dreconstruction by using Mimics 17.0 (Materialize, Leuven, Belgium). The CT and MRI images were used to develop the bony structures and soft tissues, respectively. The reconstructed CT and MRI models were combined with the positional alignment of each model using commercial software (Rapidform version 2006; 3D Systems Korea Inc., Seoul, South Korea).

The bony structures were modeled as rigid bodies (Pena et al., 2006). The articular cartilage and menisci were modeled as isotropic and transversely isotropic, respectively, with linear elastic material properties (Haut Donahue et al., 2003). Additionally, the major ligaments were modeled with nonlinear and tension-only spring elements (Kang et al., 2017c; Koh et al., 2017). The force–displacement relationship based on the functional bundles in the actual ligament anatomy is as follows:

$$ f\left(\varepsilon \right)=\left\{\begin{array}{c}\frac{k{\varepsilon}^2}{4{\varepsilon}_1},0\le \varepsilon \le 2{\varepsilon}_1\\ {}k\left(\varepsilon -{\varepsilon}_1\right),\varepsilon >2{\varepsilon}_1\\ {}0,\varepsilon <0\end{array}\right. $$

$$ \varepsilon =\frac{l-{l}_0}{l_0} $$

$$ {l}_0=\frac{l_r}{\upvarepsilon_{\mathrm{r}}+1} $$

where f(ε) denotes the current force, k denotes the stiffness, ε denotes the strain, and ε1 is assumed to be a constant (0.03). The ligament bundle slack length l0 is calculated using the reference bundle length and the reference strain in the upright reference position.

The interfaces between the articular cartilage and bones were assumed to be fully bonded. Six pairs of TF contacts between the femoral cartilage and meniscus, meniscus and tibial cartilage, and femoral cartilage and tibial cartilage were modeled for both the medial and lateral sides (Kim et al., 2015).

### Development of post-cam and TF conformity for TKA model

**Fig. 2:**
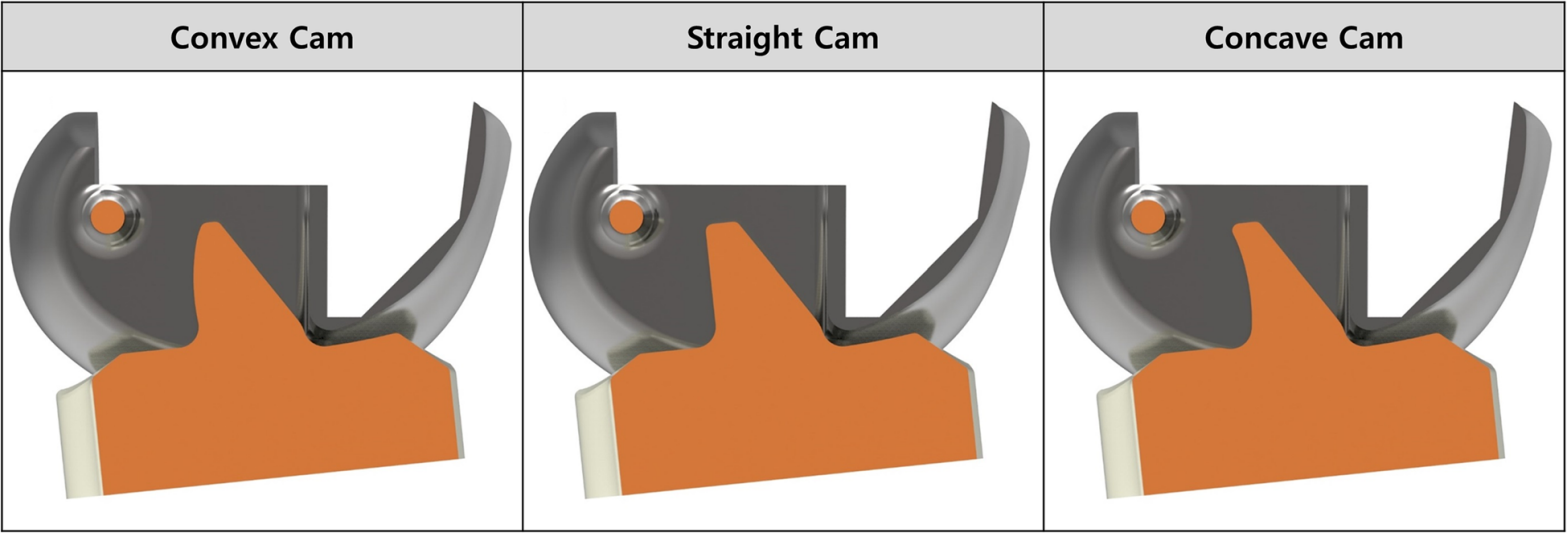
Three different custom-designed cams in the tibial component

We developed three different types of articular surface three-dimensional (3D) models for PS TKA. All post-cam designs had a curved and curved post-cam in the axial cross-sectional plane for three of articular surface for PS TKA. In the lateral view, the circle geometrical designed cam was considered the femoral component, and three different cdesigned posts (convex, straight, and concave) were considered in the tibial bearing component (Fig. 2). We fixed the post anterior-posterior position, post size (height, width, and depth), and cam position (distance from the posterior edge and height above the joint line) to solely investigate the effects of the post-cam design. We generated the conventional PS TKA Genesis II Total Knee System (Smith & Nephew Inc., Memphis, TN, USA). In addition, three different post-cam designs were applied to three different TF joint conformities.

**Fig. 3:**
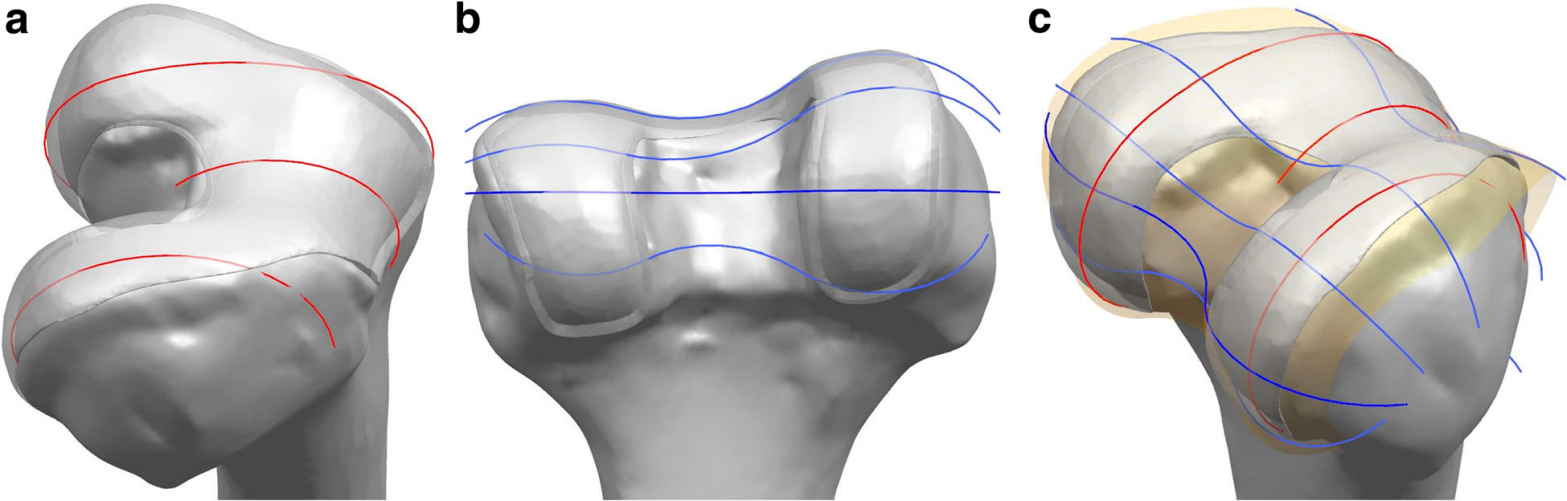
Development of customized femoral component: (**a**) three patient-specific “J” curves in sagittal planes; (**b**) patient’s anatomic curves in coronal planes; (**c**) surface geometry for femoral component using the articulating curves

We applied a previously developed design technique for a customized femoral component in this study (“ConforMIS, Inc, n.d.,” http://www.conformis.com.; Kang et al., 2017c; Koh et al., 2017). 3D reconstruction and editing of the models were performed using the Mimics and 3-Matic software (Materialise, Leuven, Belgium). Planes were introduced by the intersection of the condyles in both the sagittal and coronal planes. Intersection curves were used to extract the articulating surface geometry in both planes. The three patient-specific “J” curves for the trochlear grooves and the medial and lateral condyles from the patients’ normal articular anatomy were developed using the Unigraphics NX software (Version 7.0; Siemens PLM Software, Torrance, CA, USA) (Fig. 3). This natural condylar offset was defined as the coronal offset.

On the tibial plateau, the profile of the patient’s tibia defines the geometry of the tibial implant. In this method, the patient receives an implant that provides better tibial plateau bone coverage with reduced contact stress in the PE insert (“ConforMIS, Inc.,” http://www.conformis.com.; Harrysson et al., 2007; Kurtz et al., 2016; Steklov et al., 2010; Heever et al., 2010). Generally, the articular geometry in a customized tibial insert design is derived from the femoral component. The geometry of the medial insert is slightly more conforming than that of the lateral insert. The coronal geometry utilizes a broad radius on both condyles, and thereby employs the round-on-round principle, which was shown to reduce contact stress (Kurtz et al., 2016).

Three different tibial inserts were developed to investigate TF articular surface conformity in this study. We applied the TF conformity of a conventional PS TKA to a patient-specific TKA conformity. To achieve this, conforming design TKAs such as the Genesis II Total Knee System (Smith & Nephew Inc.) and the medial pivot design Evolution Total Knee Arthroplasty (Wright Medical Technology, Arlington, TN, USA) were selected. The conformities of the conventional TKAs were investigated by scanning them using a noncontact 3D laser scanner (COMET VZ; Steinbichler Optotechnik GmbH, Neubeuern, Germany) with an accuracy of 50 μm. Scanned point data were converted to 3D models, and scanning was repeated until the 3D model dimensions had geometrical errors of < 100 μm (Kwon et al., 2014).

**Fig. 4:**
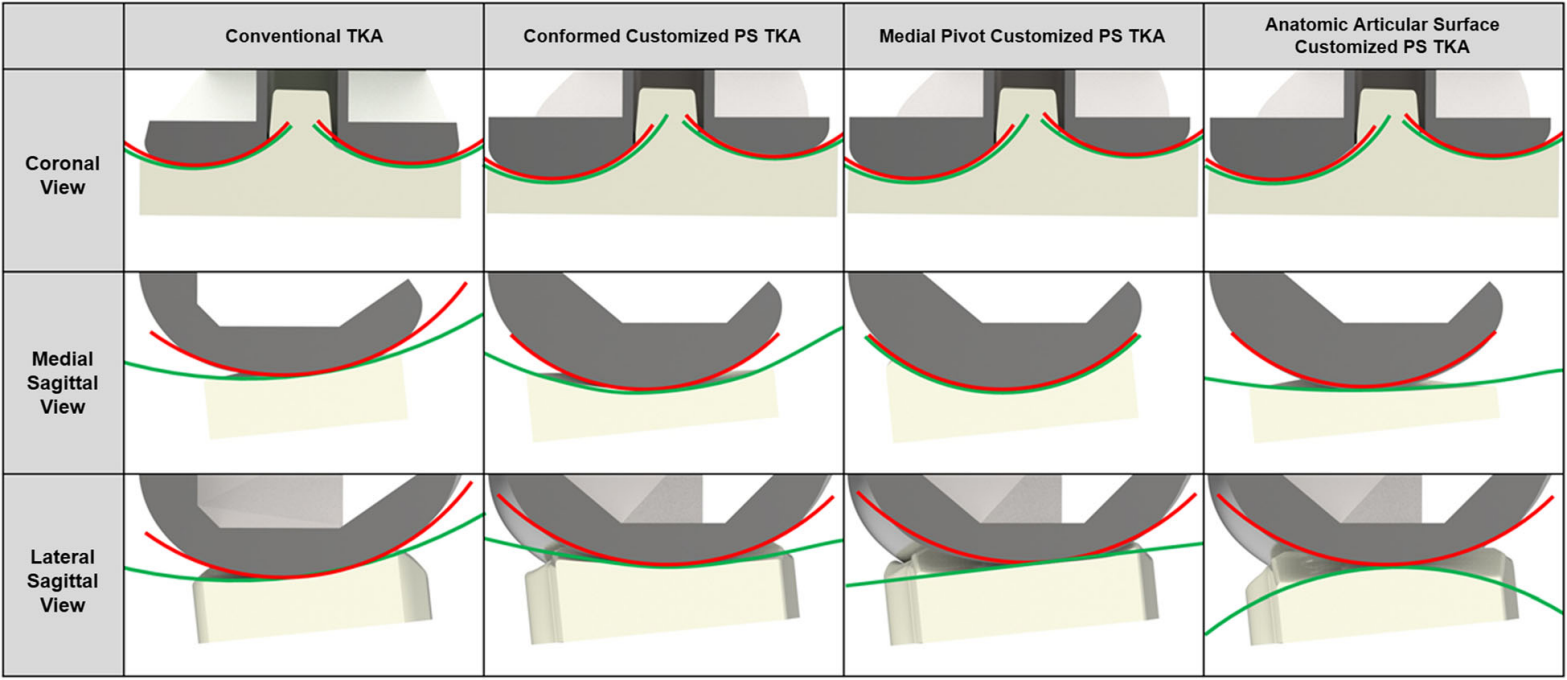
Conventional and customized PS-TKAs with different TF joint conformity designs by applying the curvature radius ratio in the coronal, medial, and lateral planes

The ratio of the curvature radius of the tibial insert to the total curvature radius of the femoral component was investigated for conformity in the coronal and sagittal planes. A tibial insert with conventional PS TKA conformity (Genesis II) and medial pivot tibial insert with medial pivot conformity (Evolution) were developed by applying the curvature radius ratio in the coronal and sagittal planes for patient-specific femoral components (Fig. 4). Additionally, an anatomy mimetic patient-specific TKA was developed in which the femoral and tibial articular surface followed the patient’s geometry (Fig. 4). There were three different patient-specific TKA designs: conformed customized PS TKA (CPS-TKA), medial pivot customized PS TKA (MPS-TKA), and anatomic articular surface customized PS TKA (APS-TKA). In addition, PS TKA was categorized into convex, straight, and concave with respect to post-cam designs as PSV-TKA, PSS-TKA, and PSC-TKA, respectively. The nine different customized and one conventional TKA models were implanted as described below.

In the neutral position, the femoral component was aligned such that the distal bone resection was perpendicular to the mechanical axis of the femur, and the anterior and posterior resections were parallel to the clinical epicondylar axis in the transverse plane. In addition, the femoral component was positioned 0°of flexion in the plane parallel to the anatomical axis of the distal femur. The tibial default alignment was rotated by 0° relative to the anterior-posterior axis, and the coronal alignment corresponded to 90° relative to the mechanical axis. In addition, the tibial component was implanted maintaining subject’s original posterior tibial slope. Contact conditions were applied between the femoral component, tibial insert, and patellar button in the TKA. The coefficient of friction between the PE and metal materials was assumed to be 0.04 for consistency with previous explicit FE models (Godest et al., 2002). The materials for the femoral component, PE, and tibial base plate were described in previous studies (Godest et al., 2002; Kang, Koh, et al., 2018a).

### Loading and boundary conditions

This FE investigation included four types of loading conditions corresponding to the loads used in the experimental study for model validation and model predictions for clinically relevant loading scenarios. With respect to the model validation, identical simulated loading protocols were applied in the experiment.

Under the first loading condition, 150 N was applied to the tibia with 30∘ and 90∘ flexion in the FE knee joint to evaluate the anterior and posterior tibial translations (Kang et al., 2016a). In addition, a second axial loading of 1150 N was applied to the model in order to obtain the contact stresses and compare them to those reported by a published FE study on the knee joint (Pena et al., 2006). Additionally, in order to validate the conventional TKA model, conservative ankle force of 50 Newton to simulate a portion of the body weight, which required the quadriceps- actuators to pull with a linearly rising force and a maximum of 600 N at 90 ^0^of flexion of the quadriceps actuators was exerted on the TKA model (Wunschel et al., 2013). In addition, during knee flexion, the quadriceps forces were always maintained identical to one another, while the hamstrings forces were kept constant at 10 N under the third loading condition (Wunschel et al., 2013). The fourth loading condition corresponded to deep-knee-bend loading to evaluate the effects of the post-cam design and TF joint conformity. A computational analysis was conducted with an anterior-posterior force applied to the femur with respect to the compressive load applied to the hip (Halloran et al., 2010; Kang et al., 2016b; Kutzner et al., 2010). A proportional–integral–derivative (PID) controller was incorporated into the computational model to control the quadriceps in a manner similar to that in a previous experiment (Kang et al., 2017a). A control system was used to calculate the instantaneous displacement of the quadriceps required to match the target flexion profile, which is the same as that in the experiment (Kang et al., 2017a). Internal–external torque and varus–valgus torque were both applied to the tibia (Halloran et al., 2010; Kang et al., 2016b; Kutzner et al., 2010).

The FE model was analyzed using ABAQUS software (version 6.11; Simulia, Providence, RI, USA). The kinematics in the TF joint and the forces on the quadriceps were calculated throughout the deep-knee-bending task. The anterior tibial translation was calculated based on Grood and Suntay’s definition of a joint coordinate system (Grood & Suntay, 1983).

## Results

### Validation of intact and conventional TKA models

**Fig. 5:**
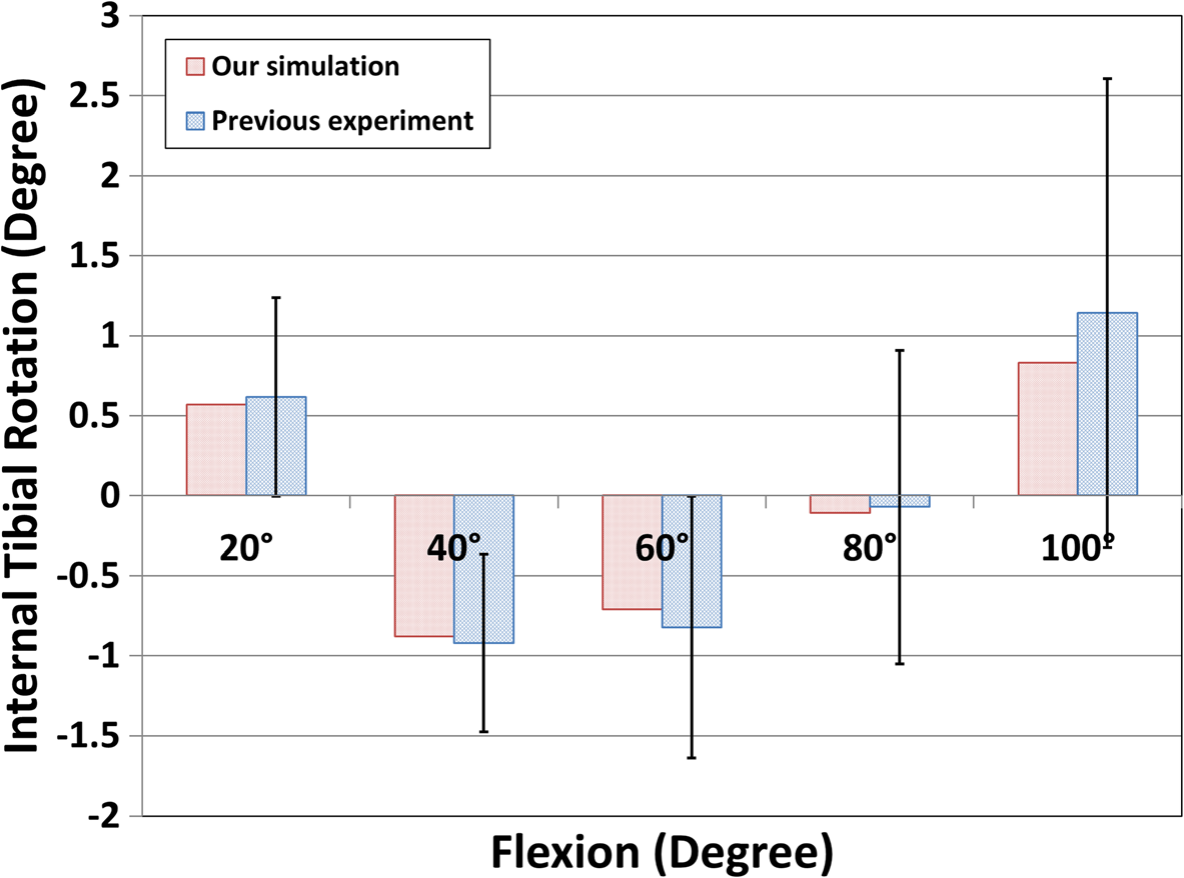
Comparison of internal tibial rotation with previous study for validation of the TKA model

For normal knee FE model validation, the results from the experiment were compared with those of the FE model’s subject. Under the loading condition with a 30° flexion, the anterior tibial translation was 2.83 mm in the experiment and 2.54 mm in the FE model, and the posterior tibial translation was 2.12 mm in the experiment and 2.18 mm in the FE model. This showed good consistency between the experimental and FE models (Kang et al., 2016a). In addition, the results were also compared with previous FE results for model validation. Contact pressures of 3.1 and 1.53 MPa were found on the medial and lateral meniscus, respectively, under an axial load of 1150 N. Both were within 4% of the contact pressures of 2.9 and 1.45 MPa similar to previous study (Pena et al., 2006). These minor differences could be caused due to variations in the geometry such as the thickness of the cartilage and meniscus between different studies. However, on an overall basis, the considerable consistency between the results of validation and reference confirmed the ability of the FE model to produce reasonable results (Pena et al., 2006). The conventional TKA FE model for the tibia was internally rotated by 0.57°, − 0.88°, − 0.71°, − 0.11°, and 0.83° under 20°, 40°, 60°, 80°, and 100° flexions, respectively (Fig. 5). There was good consistency between the simulation and previous experiment within the standard deviation under the same loading conditions applied to a prosthetic implant (Wunschel et al., 2013).

### Comparison of kinematics and quadriceps force in post-cam design and TF joint conformity

**Fig. 6:**
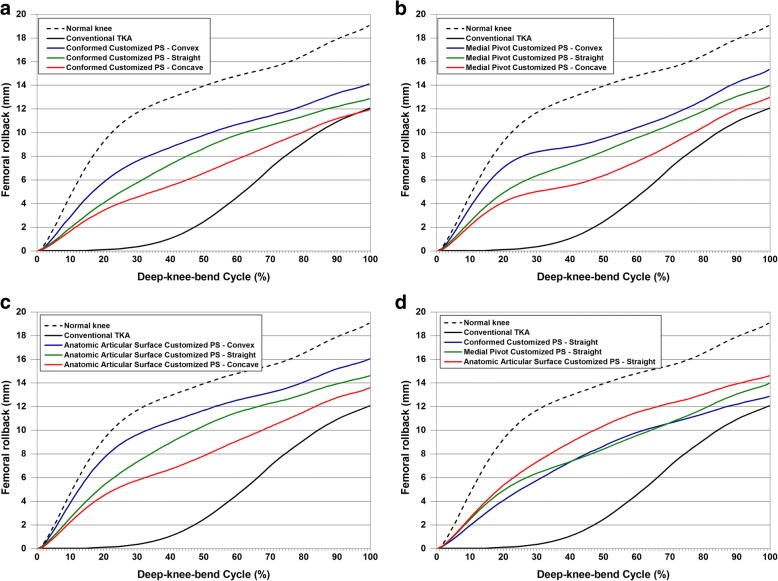
Comparison of femoral rollback in (**a**) Conformed Customized PS-TKA, (**b**) Medial Pivot Customized PS-TKA, and (**c**) Anatomic Articular Surface Customized PS-TKA with regard to different cam designs in the tibial component and (**d**) straight post designed tibial component with regard to different conformity designs in the tibial component under deep-knee-bend

**Fig. 7:**
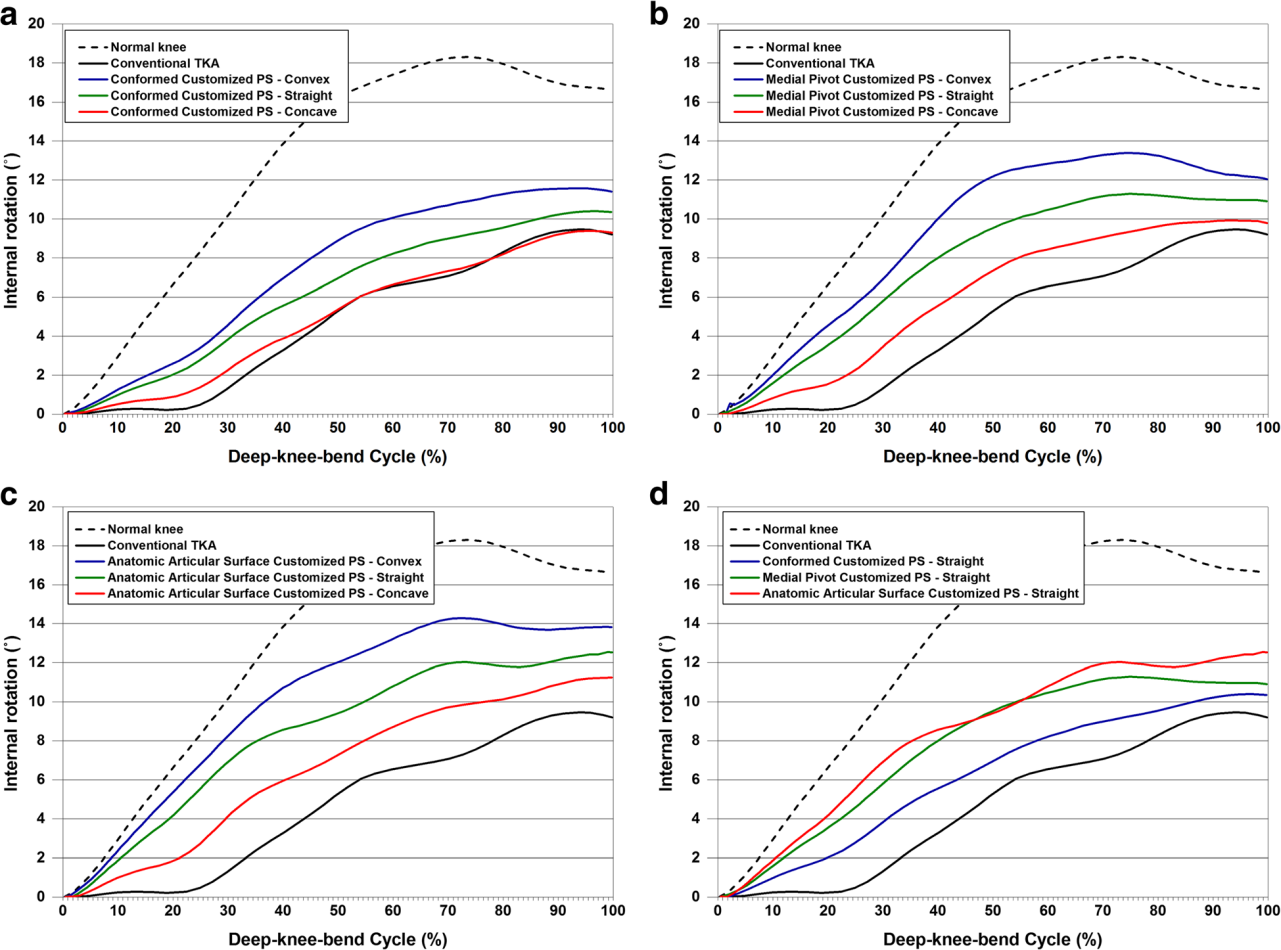
Comparison of internal rotation in (**a**) Conformed Customized PS-TKA, (**b**) Medial Pivot Customized PS-TKA, and (**c**) Anatomic Articular Surface Customized PS-TKA with regard to different cam designs in the tibial component and (**d**) straight post designed tibial component with regard to different conformity designs in the tibial component under deep-knee-bend

In all TKA models, femoral rollback decreased compared with that of a normal knee (Fig. 6). Such a trend was consistent with different post-cam designs and the conformity of the TF joint. The circle cam and convex post showed the greatest femoral rollback and the most natural-knee-like pattern regardless of TF joint conformity. In addition, the circle cam and concave post showed the lowest femoral rollback and the greatest difference from a normal knee. In terms of conformity, APS-TKA showed the greatest femoral rollback and less difference from a normal knee, and CPS-TKA showed the lowest femoral rollback and greater difference from a normal knee. Among combination designs, APS-TKA with a convex post design, i.e., APSV-TKA, showed the most natural-knee-like femoral rollback, and CPS-TKA with a concave post design, i.e., CPSC-TKA, showed the femoral rollback that has the greatest difference from that of a normal knee. However, differences between the customized TKA and a normal knee in femoral rollback were fewer than those in a conventional PS TKA. Similar to femoral rollback, all tibial internal rotation decreased in the TKA model compared with the normal knee model (Fig. 7). In particular, tibial internal rotation did not show the characteristic “screw home” motion at low flexion angles in the conventional TKA model. However, the normal knee showed a rapid increase in tibial internal rotation at a low flexion angle consistent with the “screw home” motion. Similar to femoral rollback, the circle cam and convex post showed the greatest internal rotation with less difference from the normal knee in tibial rotation. Among combination designs, APSV-TKA with a convex post design showed the most natural-knee-like internal rotation, and CPSC-TKA with concave post design showed the internal rotation with the greatest difference from a normal knee. However, differences between the customized TKA and the normal knee in internal rotation were fewer than those in a conventional PS TKA.

**Fig. 8:**
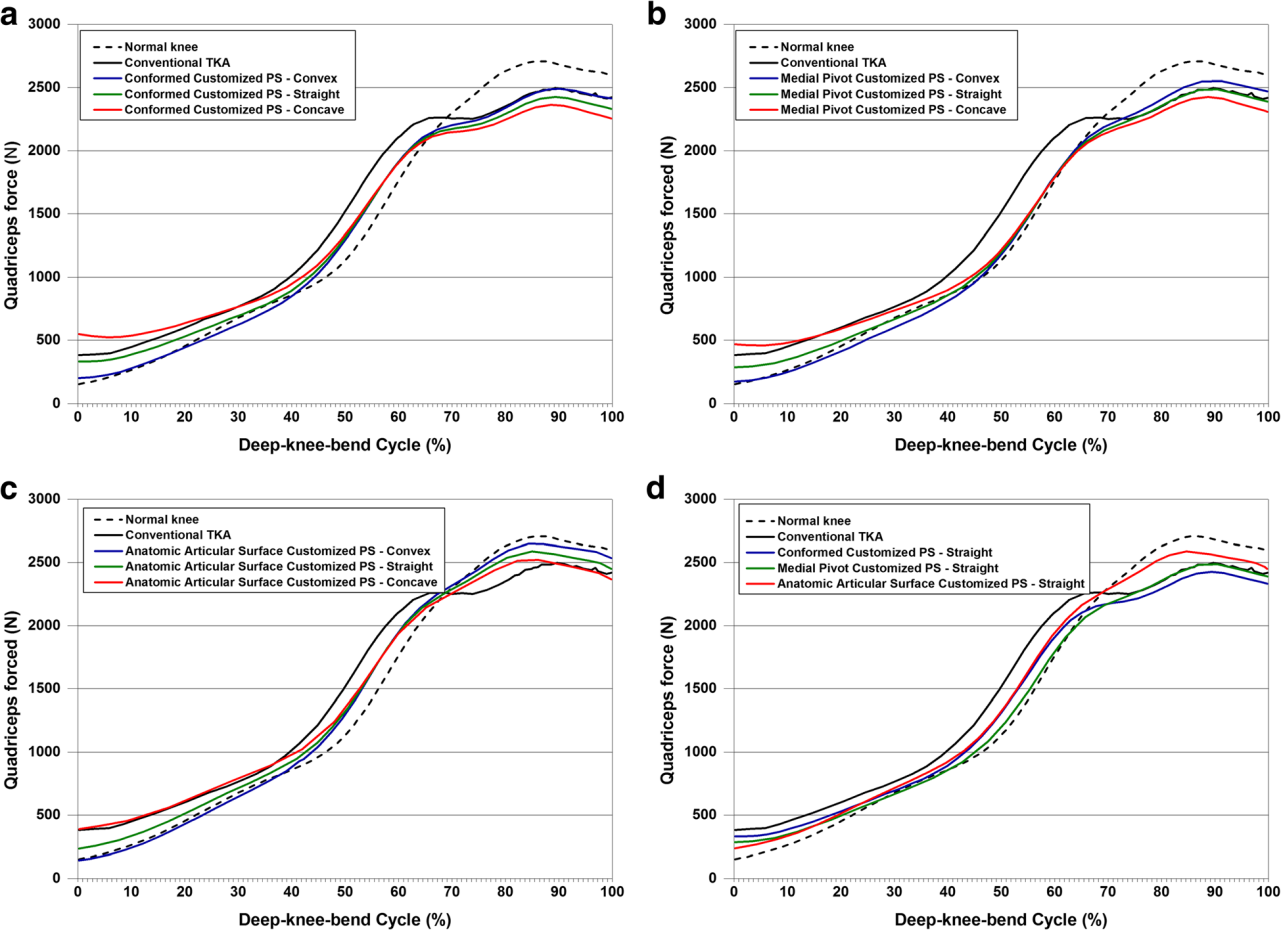
Comparison of quadriceps force in (**a**) Conformed Customized PS-TKA, (**b**) Medial Pivot Customized PS-TKA, and (**c**) Anatomic Articular Surface Customized PS-TKA with regard to different cam designs in the tibial component and (**d**) straight post designed tibial component with regard to different conformity designs in the tibial component under deep-knee-bend

All TKAs required greater and lower quadriceps force when compared with the normal knee for low and high flexions, respectively (Fig. 8). Similar to kinematics, this difference from the normal knee was the greatest in the conventional TKA. Upon combining the post-cam design and conformity, similar to kinematics, APSV-TKA with a convex post design showed the most natural-knee-like quadriceps force, and CPSC-TKA with a concave post design showed the quadriceps force that had the greatest difference from a normal knee.

## Conclusion & discussion

The most important finding of this study was that the circle cam and convex post showed the greatest femoral rollback and tibial internal rotation with the most natural-knee-like kinematics regardless of TF joint conformity. In addition, the design that described TF joint anatomy showed the most natural-knee-like kinematics. Upon combining the post-cam design and TF joint conformity, the post-cam design with TF joint conformity that described the anatomy, circle cam, and convex post showed the most natural-knee-like kinematics.

To test aforementioned hypothesis, we developed a 3D nonlinear FE model of the knee Joint with nine different customized TKA and one conventional TKA model. The intact knee and conventional TKA model followed a series of rigorous validation steps. There was consistency with previous experiments and experiments using the FE model’s subject. Therefore, the post-cam design and TF joint conformity models used in this study and the following analysis are considered reasonable. To the best of our knowledge, this study is the first to investigate the effects of different post-cam designs and TF joint conformity geometry combinations on kinematics and quadriceps force.

Previous studies support our findings indirectly. Lu et al. showed that a posterior translation of 6.5 ± 2.1 mm of the femur occurs in normal knees by using single-plane fluoroscopic images and CT bone data (Lu et al., 2008). As compared with the natural kinematics of our simulation, kinematics appears to be within the range observed for normal knees. Therefore, the values from the normal knee kinematic simulation are similar to those of the previous study. In our simulation, it was observed that femoral rollback and tibial internal rotation decreased if TKA was applied. This trend was similar to the findings in a previous study (Varadarajan et al., 2009).

Many previous studies showed low contact stress and improved kinematics for curved and curved post-cam designs in the axial cross-sectional plane (Huang et al., 2006; Lin et al., 2011; Watanabe et al., 2017). Therefore, curved and curved designs in the axial cross-sectional plane were fixed in this study. Our results showed that the circle cam and convex post in sagittal plane provided the greatest femoral rollback and internal rotation. This trend was also similar to that of a previous study (Fallahiarezoodar et al., 2014). Femoral rollback was 11.9 mm at a 120° flexion in the simulation and 10.6 mm in a previous study for CPS-TKA with a circle cam and convex post in which conventional conformity was applied (Fallahiarezoodar et al., 2014). In addition, the design that described the patient’s anatomy from the TF joint conformity showed the most natural-knee-like kinematics. Our results proved that the articular geometry of the normal knee also plays an important role in guiding knee motion. In particular, while the medial tibial plateau is concave anteroposteriorly, the lateral tibial plateau is convex (Amiri et al., 2006; Smith et al., 2003). Similarly, while the medial surface of the intercondylar eminence is straight, the lateral surface is convex (Amiri et al., 2006; Smith et al., 2003). Consequently, the lateral condyle moves on a curved path around the eminence, whereas the medial condyle experiences a “ball-in-socket” motion (Amiri et al., 2006; Smith et al., 2003). Therefore, loss of this asymmetry between the geometry of the medial and lateral tibial plateaus after TKA may cause changes in tibial rotation (Walker & Sathasivam, 2000).

Additionally, the interaction between the femoral TKA component and the posterior lip of the tibial PE component likely restricts femoral rollback (Bull et al., 2008; G. Li et al., 2006). The maximum tibial internal rotation reached approximately 12.5° in APSV-TKA throughout the knee flexion in our simulation. A previous study showed that tibial rotation reached approximately 8° throughout the knee flexion (Lin et al., 2011). A previous in vivo study reported that the maximum tibial rotation of a contemporary PS knee prosthesis (Legacy LPS-Flex, Zimmer, Warsaw, IN, USA) averaged approximately 5° (Cates et al., 2008). This trend was different from our results. However, the magnitude of tibial rotation was smaller in the Legacy LPS-Flex TKA because of the conformity of the tibial plateau and femoral condyle. The tibial plateau of the Legacy LPS-Flex TKR is more dish like in shape in the coronal plane, which may reduce contact stress of the TF joint with rotational malalignment (Liau et al., 2002). However, this geometry could produce a greater constraint on the axial motion of the femur relative to the tibia.

Notably, all TKRs required more quadriceps force than the normal knee at low flexion, and less at high flexion. Theoretically, a TKA design with the center of the flexion located relatively more posteriorly can result in additional lengthening of the quadriceps lever arm. A previous study showed that a longer lever arm reduces the tension on the quadriceps during a knee extension, especially at flexion angles that typically generate high knee moments (D’Lima et al., 2001).

Our result showed that, after TKA, the knee required more quadriceps force under a high flexion angle. These results contradicted those of a previous study that observed post-operative gait adaptations such as “quadriceps avoidance” in TKA patients (K. Li et al., 2013). However, the study reported that the TKA patients compensated for this deficiency by leaning their trunks forward. Our FE model showed different results for the quadriceps force in the PID controlled flexion, and the trunk could not guarantee the same flexion. In addition, more and less quadriceps force was required in the TKA model for low and high flexions, respectively. This is similar to a cadaveric experiment under deep-knee-bending (D’Lima et al., 2001). A previous study showed that the quadriceps ratios from isokinetic testing of these three prosthesis design groups were greater than those of the healthy group (Walker et al., 2009).

Despite advances in TKA design, many patients still experience limited kinematics in their normal daily function, leading to reduced patient satisfaction (Banks et al., 2003; Most et al., 2005). During our deep knee bend simulation, TKA showed substantial reduction in internal tibial rotation compared to normal knee. This trend was similar to previous study (Varadarajan et al., 2009). In particularly, conventional did not show the characteristic screw home motion low flexion angle. Conventional TKA has design that does not describe present normal knee anatomy thus it was found more. This is evident in the Asian population, where an average individual is required to adopt special posture positions in their daily activities, such as a gait or a deep-knee-bend (Hefzy et al., 1998). To overcome this, several TKA designs proposed new features such as changing the posterior sagittal femoral geometry to prevent the occurrence of PE edge loading and increase the articulation curvature during deep-knee flexion (Zelle et al., 2009). However, greater flexion angles have led to a high poly wear rate, which results in an increased number of surgeries required for patients during their lifetime.

In term of clinical relevance, our simulation showed not only importance of mimetic TKA design using anatomy, but also change of kinematics with respect to post-cam design However, perfect natural knee kinematics could not be restored although anatomy was perfectly applied and post-cam design was applied in different way. It is because of absence of anterior cruciate ligament. Recent study showed that ACL-substituting implants could be a valuable treatment option capable of overcoming the limitations of contemporary TKA, particularly when retaining the native ACL is not feasible or is challenging. However, our results showed that a post-cam design with an anatomy mimetic articular surface, circle cam, and convex post showed the most natural-knee-like mechanics (Zumbrunn et al., 2018).

Although this study is the first to compare a combination of post-cam designs and TF joint conformities in order to provide natural-knee-like kinematics, some limitations should be acknowledged so that the findings of this study are not overstated. First, although a deep-knee-bending simulation was performed, additional simulations related to more demanding activities (e.g., rising from a chair, sitting and climbing, and descending stairs) are required in the future for a more reliable investigation. However, the simulation was performed under deep-knee-bending motions because such motions include both a wide range of flexion/extension and a significant muscular endeavor around the knee joint. Second, the results cannot be utilized instead of clinical outcomes, and they do not consider patient satisfaction because of their sole correspondence to the outcomes of computational analyses. However, the main factor analyzed in the present study corresponds to the main investigating components in evaluating the biomechanical effect of computational biomechanics (Fitzpatrick et al., 2012; Haut Donahue et al., 2003; Huang et al., 2006; Kang et al., 2016a; Kang et al., 2017b; Kang et al., 2017c; Kang et al., 2018b; Kiapour et al., 2014; Kim et al., 2015; Koh et al., 2017; Lin et al., 2011; Pena et al., 2006; Watanabe et al., 2017). Third, although the material properties and attachment points for the ligaments used in the model were assumed based on previously published studies, considerable variability exists. However, our objective was not to determine the quantitative values for muscle and ligament forces but to determine the effects of variability in the post-cam design and TF joint conformity on our variables of interest.

In conclusion, we investigated normal knee kinematics with many combinations of post-cam design and TF joint conformity under a deep-knee-bend simulation. Our results showed that a post-cam design with an anatomy mimetic articular surface, circle cam, and convex post showed the most natural-knee-like mechanics. This study suggested that post-cam design and TF surface conformity should be considered in combination for conventional and customized TKA.

## Abbreviations

3D: Three-dimensional
APS-TKA: Articular surface customized PS TKA
CPS-TKA: Customized PS TKA
CR: Cruciate-retaining
CT: Computed tomography
FE: Finite element
MPS-TKA: Medial pivot customized PS TKA
PCL: Posterior cruciate ligament
PE: Polyethylene
PF: Patellofemoral
PS: Posterior-stabilized
TF: Tibiofemoral
TKA: Total knee arthroplasty

## References

[CR1] Amiri S, Cooke D, Kim IY, Wyss U (2006). Mechanics of the passive knee joint. Part 1: the role of the tibial articular surfaces in guiding the passive motion. Proc Inst Mech Eng H.

[CR2] Argenson JN, Scuderi GR, Komistek RD, Scott WN, Kelly MA, Aubaniac JM (2005). In vivo kinematic evaluation and design considerations related to high flexion in total knee arthroplasty. J Biomech.

[CR3] Arnout N, Vanlommel L, Vanlommel J, Luyckx JP, Labey L, Innocenti B, Bellemans J (2015). Post-cam mechanics and tibiofemoral kinematics: a dynamic in vitro analysis of eight posterior-stabilized total knee designs. Knee Surg Sports Traumatol Arthrosc.

[CR4] Banks S, Bellemans J, Nozaki H, Whiteside LA, Harman M, Hodge WA (2003) Knee motions during maximum flexion in fixed and mobile-bearing arthroplasties. Clin Orthop Relat Res (410):131–138. 10.1097/01.blo.0000063121.39522.19 10.1097/01.blo.0000063121.39522.19 12771823

[CR5] Blaha JD (2004). The rationale for a total knee implant that confers anteroposterior stability throughout range of motion. J Arthroplast.

[CR6] Bull AM, Kessler O, Alam M, Amis AA (2008). Changes in knee kinematics reflect the articular geometry after arthroplasty. Clin Orthop Relat Res.

[CR7] Cates HE, Komistek RD, Mahfouz MR, Schmidt MA, Anderle M (2008). In vivo comparison of knee kinematics for subjects having either a posterior stabilized or cruciate retaining high-flexion total knee arthroplasty. J Arthroplast.

[CR8] ConforMIS, (2018) Inc. n.d. http://www.conformis.com.

[CR9] D'Lima DD, Poole C, Chadha H, Hermida JC, Mahar A, Colwell CW Jr (2001) Quadriceps moment arm and quadriceps forces after total knee arthroplasty. Clin Orthop Relat Res (392):213–220 10.1097/00003086-200111000-00026 11716385

[CR10] Fallahiarezoodar A, Abdul Kadir MR, Alizadeh M, Naveen SV, Kamarul T (2014). Geometric variable designs of cam/post mechanisms influence the kinematics of knee implants. Knee Surg Sports Traumatol Arthrosc.

[CR11] Fitzpatrick CK, Clary CW, Rullkoetter PJ (2012). The role of patient, surgical, and implant design variation in total knee replacement performance. J Biomech.

[CR12] Godest AC, Beaugonin M, Haug E, Taylor M, Gregson PJ (2002). Simulation of a knee joint replacement during a gait cycle using explicit finite element analysis. J Biomech.

[CR13] Grood ES, Suntay WJ (1983). A joint coordinate system for the clinical description of three-dimensional motions: application to the knee. J Biomech Eng.

[CR14] Halloran JP, Clary CW, Maletsky LP, Taylor M, Petrella AJ, Rullkoetter PJ (2010). Verification of predicted knee replacement kinematics during simulated gait in the Kansas knee simulator. J Biomech Eng.

[CR15] Harrysson OL, Hosni YA, Nayfeh JF (2007). Custom-designed orthopedic implants evaluated using finite element analysis of patient-specific computed tomography data: femoral-component case study. BMC Musculoskelet Disord.

[CR16] Haut Donahue TL, Hull ML, Rashid MM, Jacobs CR (2003). How the stiffness of meniscal attachments and meniscal material properties affect tibio-femoral contact pressure computed using a validated finite element model of the human knee joint. J Biomech.

[CR17] Hefzy MS, Kelly BP, Cooke TD (1998). Kinematics of the knee joint in deep flexion: a radiographic assessment. Med Eng Phys.

[CR18] Huang CH, Liau JJ, Huang CH, Cheng CK (2006). Influence of post-cam design on stresses on posterior-stabilized tibial posts. Clin Orthop Relat Res.

[CR19] Insall JN, Hood RW, Flawn LB, Sullivan DJ (1983). The total condylar knee prosthesis in gonarthrosis. A five to nine-year follow-up of the first one hundred consecutive replacements. J Bone Joint Surg Am.

[CR20] Kang KT, Kim SH, Son J, Lee YH, Chun HJ (2016). Computational model-based probabilistic analysis of in vivo material properties for ligament stiffness using the laxity test and computed tomography. J Mater Sci Mater Med.

[CR21] Kang KT, Koh YG, Jung M, Nam JH, Son J, Lee YH, Kim SH (2017). The effects of posterior cruciate ligament deficiency on posterolateral corner structures under gait- and squat-loading conditions: a computational knee model. Bone Joint Res.

[CR22] Kang KT, Koh YG, Son J, Kim SJ, Choi S, Jung M, Kim SH (2017). Finite element analysis of the biomechanical effects of 3 posterolateral corner reconstruction techniques for the knee joint. Arthroscopy.

[CR23] Kang KT, Koh YG, Son J, Kwon OR, Baek C, Jung SH, Park KK (2016). Measuring the effect of femoral malrotation on knee joint biomechanics for total knee arthroplasty using computational simulation. Bone Joint Res.

[CR24] Kang KT, Koh YG, Son J, Kwon OR, Lee JS, Kwon SK (2018). Influence of increased posterior Tibial slope in Total knee arthroplasty on knee joint biomechanics: a computational simulation study. J Arthroplast.

[CR25] Kang KT, Son J, Kwon OR, Koh YG (2017). Malpositioning of prosthesis: patient-specific Total knee arthroplasty versus standard off-the-shelf Total knee arthroplasty. J Am Acad Orthop Surg Glob Res Rev.

[CR26] Kang KT, Son J, Suh DS, Kwon SK, Kwon OR, Koh YG (2018). Patient-specific medial unicompartmental knee arthroplasty has a greater protective effect on articular cartilage in the lateral compartment: a finite element analysis. Bone Joint Res.

[CR27] Kiapour A, Kiapour AM, Kaul V, Quatman CE, Wordeman SC, Hewett TE, Goel VK (2014). Finite element model of the knee for investigation of injury mechanisms: development and validation. J Biomech Eng.

[CR28] Kim YS, Kang KT, Son J, Kwon OR, Choi YJ, Jo SB, Koh YG (2015). Graft extrusion related to the position of allograft in lateral meniscal allograft transplantation: biomechanical comparison between Parapatellar and Transpatellar approaches using finite element analysis. Arthroscopy.

[CR29] Koh YG, Son J, Kwon OR, Kwon SK, Kang KT (2018) Tibiofemoral conformity variation offers changed kinematics and wear performance of customized posterior-stabilized total knee arthroplasty. Knee Surg Sports Traumatol Arthrosc. 10.1007/s00167-018-5045-9 10.1007/s00167-018-5045-9 29974167

[CR30] Koh YG, Son J, Kwon SK, Kim HJ, Kwon OR, Kang KT (2017). Preservation of kinematics with posterior cruciate-, bicruciate- and patient-specific bicruciate-retaining prostheses in total knee arthroplasty by using computational simulation with normal knee model. Bone Joint Res.

[CR31] Kumar N, Yadav C, Raj R, Yadav S (2015). Fracture of the polyethylene tibial post in a posterior stabilized knee prosthesis: a case report and review of literature. J Orthop.

[CR32] Kurtz WB, Slamin JE, Doody SW (2016) Bone preservation in a novel patient specific Total knee replacement. Reconstructive Review 6(1):19–23.

[CR33] Kutzner I, Heinlein B, Graichen F, Bender A, Rohlmann A, Halder A, Bergmann G (2010). Loading of the knee joint during activities of daily living measured in vivo in five subjects. J Biomech.

[CR34] Kwon OR, Kang KT, Son J, Kwon SK, Jo SB, Suh DS, Koh YG (2014). Biomechanical comparison of fixed- and mobile-bearing for unicomparmental knee arthroplasty using finite element analysis. J Orthop Res.

[CR35] Lachiewicz PF (2011). How to treat a tibial post fracture in total knee arthroplasty? A systematic review. Clin Orthop Relat Res.

[CR36] Li G, Most E, Sultan PG, Schule S, Zayontz S, Park SE, Rubash HE (2004). Knee kinematics with a high-flexion posterior stabilized total knee prosthesis: an in vitro robotic experimental investigation. J Bone Joint Surg Am.

[CR37] Li G, Suggs J, Hanson G, Durbhakula S, Johnson T, Freiberg A (2006). Three-dimensional tibiofemoral articular contact kinematics of a cruciate-retaining total knee arthroplasty. J Bone Joint Surg Am.

[CR38] Li K, Ackland DC, McClelland JA, Webster KE, Feller JA, de Steiger R, Pandy MG (2013). Trunk muscle action compensates for reduced quadriceps force during walking after total knee arthroplasty. Gait Posture.

[CR39] Liau JJ, Cheng CK, Huang CH, Lo WH (2002). The effect of malalignment on stresses in polyethylene component of total knee prostheses--a finite element analysis. Clin Biomech (Bristol, Avon).

[CR40] Lin KJ, Huang CH, Liu YL, Chen WC, Chang TW, Yang CT, Cheng CK (2011). Influence of post-cam design of posterior stabilized knee prosthesis on tibiofemoral motion during high knee flexion. Clin Biomech (Bristol, Avon).

[CR41] Liu YL, Chen WC, Yeh WL, McClean CJ, Huang CH, Lin KJ, Cheng CK (2012). Mimicking anatomical condylar configuration into knee prosthesis could improve knee kinematics after TKA - a computational simulation. Clin Biomech (Bristol, Avon).

[CR42] Lu TW, Tsai TY, Kuo MY, Hsu HC, Chen HL (2008). In vivo three-dimensional kinematics of the normal knee during active extension under unloaded and loaded conditions using single-plane fluoroscopy. Med Eng Phys.

[CR43] Mauerhan DR (2003). Fracture of the polyethylene tibial post in a posterior cruciate-substituting total knee arthroplasty mimicking patellar clunk syndrome: a report of 5 cases. J Arthroplast.

[CR44] Most E, Li G, Sultan PG, Park SE, Rubash HE (2005). Kinematic analysis of conventional and high-flexion cruciate-retaining total knee arthroplasties: an in vitro investigation. J Arthroplasty.

[CR45] Pandit H, Ward T, Hollinghurst D, Beard DJ, Gill HS, Thomas NP, Murray DW (2005). Influence of surface geometry and the cam-post mechanism on the kinematics of total knee replacement. J Bone Joint Surg Br.

[CR46] Pena E, Calvo B, Martinez MA, Palanca D, Doblare M (2006). Why lateral meniscectomy is more dangerous than medial meniscectomy. A finite element study. J Orthop Res.

[CR47] Smith PN, Refshauge KM, Scarvell JM (2003). Development of the concepts of knee kinematics. Arch Phys Med Rehabil.

[CR48] Steklov N, Slamin J, Srivastav S, D'Lima D (2010). Unicompartmental knee resurfacing: enlarged tibio-femoral contact area and reduced contact stress using novel patient-derived geometries. Open Biomed Eng J.

[CR49] Van Den Heever DJ, Scheffer C, Erasmus PJ, Dillon EM (2010). Contact stresses in a patient-specific unicompartmental knee replacement. Conf Proc IEEE Eng Med Biol Soc.

[CR50] Varadarajan KM, Harry RE, Johnson T, Li G (2009). Can in vitro systems capture the characteristic differences between the flexion-extension kinematics of the healthy and TKA knee?. Med Eng Phys.

[CR51] Victor J, Bellemans J (2006). Physiologic kinematics as a concept for better flexion in TKA. Clin Orthop Relat Res.

[CR52] Walker PS, Arno S, Borukhoy I, Bell CP (2015). Characterising knee motion and laxity in a testing machine for application to total knee evaluation. J Biomech.

[CR53] Walker PS, Lowry MT, Kumar A (2014). The effect of geometric variations in posterior-stabilized knee designs on motion characteristics measured in a knee loading machine. Clin Orthop Relat Res.

[CR54] Walker PS, Sathasivam S (2000). Design forms of total knee replacement. Proc Inst Mech Eng H.

[CR55] Walker PS, Sussman-Fort JM, Yildirim G, Boyer J (2009). Design features of total knees for achieving normal knee motion characteristics. J Arthroplast.

[CR56] Watanabe T, Koga H, Horie M, Katagiri H, Sekiya I, Muneta T (2017). Post-cam Design and contact stress on Tibial posts in posterior-stabilized Total knee prostheses: comparison between a rounded and a squared design. J Arthroplast.

[CR57] Wunschel M, Leasure JM, Dalheimer P, Kraft N, Wulker N, Muller O (2013). Differences in knee joint kinematics and forces after posterior cruciate retaining and stabilized total knee arthroplasty. Knee.

[CR58] Zelle J, Van der Zanden AC, De Waal Malefijt M, Verdonschot N (2009). Biomechanical analysis of posterior cruciate ligament retaining high-flexion total knee arthroplasty. Clin Biomech (Bristol, Avon).

[CR59] Zumbrunn T, Duffy MP, Rubash HE, Malchau H, Muratoglu OK, Varadarajan KM (2018). ACL substitution may improve kinematics of PCL-retaining total knee arthroplasty. Knee Surg Sports Traumatol Arthrosc.

